# Sharing on Facebook and Face-to-Face What Others Do or Approve: Word-of-Mouth Driven by Social Norms

**DOI:** 10.3389/fpsyg.2021.712253

**Published:** 2021-10-04

**Authors:** Mingyue Zhang, Jingyi Lu, William K. Hallman

**Affiliations:** ^1^Asia Europe Business School, Faculty of Economics and Management, East China Normal University, Shanghai, China; ^2^School of Psychology and Cognitive Science, East China Normal University, Shanghai, China; ^3^Department of Human Ecology, Rutgers University, New Brunswick, NJ, United States

**Keywords:** electronic word-of-mouth, social media, reference group, descriptive norms, injunctive norms, psychological distance

## Abstract

Information sharing on social media [i.e., electronic word-of-mouth, (eWOM) and face-to-face word-of-mouth (fWOM)] plays an important role in message dissemination. This study investigates the effectiveness of group norms in motivating eWOM and fWOM. Drawing upon the psychological distance and construal level literature, this study tests the impact of group norms, the interaction effect of norms type (descriptive vs. injunctive norms), and the group distance on eWOMand fWOM. Based on one field study and three laboratory experiments, this study finds that normative cues in messages are impactful in driving WOM and the impact becomes especially stronger when the psychological distance of the social group is congruent with that of norms type tied to the group. Specifically, an interaction effect emerges, such as distant (close) group injunctive (descriptive) norms, are more impactful in driving WOM than close (distant) group injunctive (descriptive) norms. Contrary to the conventional wisdom that a close group has greater impacts than distant groups in terms of social influence, this study shows that messages with distant groups are more (or at least equally) likely to be shared than with a close group when tied with injunctive norms. The findings suggest that group norms are perceived to be more relevant when there is a match between the psychological distance of the social group and the norms type tied to the group.

## Introduction

People are increasingly getting news from social media websites such as Facebook and Twitter. According to a recent survey on news use across social media platforms, approximately 55% of U.S. adults read news on social media. Among those, 73% of users of Facebook get news on the site, as do 71% of users of Twitter and 62% of users of Reddit (Pew Research Center, 2019). Given the impacts of social media in terms of information sharing and distribution, it is of significance to make clear the factors that impact the intentions of the consumers to share news articles on social media in the form of electronic word-of-mouth (eWOM).

Word-of-mouth has long been recognized as an influential tool for disseminating messages and driving consumer communication (Arndt, 1967; Kaplan and Haenlein, 2011; Berger, 2014). The emergence of social networking sites (SNS) shifts information transmission from traditional one-on-one communication to broadcast-like online distribution. This makes WOM communication on SNS (eWOM) even more efficient and powerful than ever before (Flanagin, 2001; Dellarocas, 2003; Norman and Russell, 2006; Ho and Dempsey, 2010). A previous study has shown that stories that are useful, practical, surprising, interesting, novel, or even controversial are more likely to be shared by online users (Bakshy et al., 2011; Berger and Milkman, 2012; Chen and Berger, 2013). In addition, messages inciting emotions such as amusement or anger stimulate WOM, whereas depressing messages hinder it (Berger, 2011; Berger and Schwartz, 2011). Despite the large body of research that has examined factors driving WOM with respect to message content, the impact of research on normative cues on WOM is scant.

Normative cues are prevalent in the news, articles, and even personal messages. Group norms such as what most people typically do (descriptive norms) or approve (injunctive norms) suggest the prevalence of certain behaviors or opinions among a particular group of people. It is not uncommon to spot people sharing word-of-mouth messages about what others do or believe. Those behaviors or opinions are typically in the form of group norms and are adopted by the majority of a population.

Normative cues are a significant predictor of the usefulness of WOM in the context of online customer reviews (Cheung et al., 2007; Filieri et al., 2015); however, it remains unclear whether normative cues impact the likelihood of WOM communication (i.e., are messages with normative cues more likely to be shared than those without?). This study examines the impact of normative cues-featured messages on WOM communication intentions by associating descriptive or injunctive norms with a social group in a news article and tests whether such normative cues motivate willingness to share the article on social media (i.e., Facebook) and face-to-face (fWOM). Furthermore, we try to demonstrate that, rather than functioning alone, the type of norms (descriptive vs. injunctive norms) interacts with the perceived psychological distance of the social group when impacting WOM communication. We draw upon the literature on psychological distance and construal level and predict that consumers would be more willing to share messages featuring group norms when the psychological distance of the social group and the type of norms are congruent than when incongruent. The central theoretical purpose of this study is to examine how the WOM behavior of consumers varies as a function of the social group distance and type of norms tied to that group.

### Normative Cues and WOM

Research on the adoption of WOM with respect to online customer reviews shows that normative cues had a great impact on the adoption of the consumers of the recommendations of others. Crowd opinions such as the overall product ranking and customer rating reflect the aggregate evaluations of the product from a number of reviewers. These normative cues composed of the opinions of a majority represent the wisdom of the crowd and, are thus perceived to be more trustworthy, objective, and helpful than single reviews (Borgida and Nisbett, 1977; Bar-Hillel, 1980; Nisbett and Ross, 1980). Thus, positive overall ranking and customer ratings could increase the perceived information diagnosticity of online customer reviews, which therefore improves the information adoption of consumers (Filieri, 2015). Similarly, research by Cheung et al. (2007) suggested that normative information on online consumer discussion forums, such as customer rating and recommendation consistency, also impacts the credibility of online customer reviews, leading to increased adoption of those recommendations.

As another form of crowd wisdom, group norms reflect what most members of a social group typically do (descriptive norms) or approve (injunctive norms) (Cialdini and Trost, 1998). Group norms influence the behaviors of people in a dramatic way. They provide a benchmark informing people what the effective or adaptive behaviors are in a particular situation, and motivate action by promising social rewards and punishment (informal sanctions) (Jacobson et al., 2010). People especially attend to the norms when they are new to the environment or uncertain about their decisions. These findings suggest that messages with group norms information should be perceived as more important and credible. In other words, the normative cues in messages act as an implicit way of showing its importance and credibility. In this study, we focus on news articles that incorporate information about group norms and speculate that normative cues in the news article would increase the willingness of consumers to share. Based on this argument, we hypothesize that:

**H1**: Messages with group norms are more likely to be shared on social media than messages without group norms information.

### Type of Norms and WOM

When describing group norms, Cialdini et al. (1990) and Cialdini et al. (1991) suggested that norms reflect two kinds of conceptions, such as things people should do (injunctive norms) and things people actually do (descriptive norms; Cialdini and Trost, 1998; Donald and Cooper, 2001; see Rivis and Sheeran, 2003). Specifically, descriptive norms reflect the perception of what behaviors most group members are actually performing, whereas injunctive norms reflect the perception of what most group members approve or disapprove. Therefore, injunctive norms typically serve as a standard in the form of the attitudes of the majority of the group toward a particular issue (e.g., negative attitudes toward alcohol abuse among most college students), whereas descriptive norms prescribe a particular behavior that the group majority typically performs (e.g., most college students typically finish their credits within 4 years).

Prior research on the social influence of group norms suggests that different types of norms (descriptive vs. injunctive norms) exhibit different impacts on the behavior of people. Studies have shown that a provincial group descriptive norm is more impactful than a global group descriptive norm in persuading people to engage in sustainable consumption (Goldstein et al., 2008; Ryoo et al., 2017). More recent research has shown that descriptive norms have a greater impact on the decisions of an individual, whereas injunctive norms have a greater influence on recommendations to others (Zou and Savani, 2019).

In terms of WOM (opinions/recommendations of others) adoption, prior research has shown that different types of norms (descriptive vs. injunctive norms) exhibit different impacts on the adoption of a WOM recommendation. Zhao and Xie (2011) found that increased temporal distance from the purchasing event could improve the acceptance of consumers of recommendations from distant others by increasing the perceived relevance of the recommendation. Echoing this research, another study showed that increased temporal distance of the implementation of a policy improved the susceptibility of consumers to group voting opinions (i.e., either approval or disapproval of the policy) (Ledgerwood and Callahan, 2012). Closer spatial distance of a reference group has been shown to make consumers more likely to adopt descriptive norms and to conform to towel-recycling behavior (Goldstein et al., 2008; Ryoo et al., 2017). It is noteworthy that consumers are more likely to accept the opinions of distant others on a distant future event (Zhao and Xie, 2011) whereas, they rely more on the behaviors of close others to decide on a temporally close event (Goldstein et al., 2008; Ryoo et al., 2017). Although many studies have investigated WOM adoption with regards to normative influence (Goldstein et al., 2008; Zhao and Xie, 2011; Ledgerwood and Callahan, 2012; Ryoo et al., 2017), little is known about what causes such differences between descriptive norms vs. injunctive norms in terms of their impacts on the adoption of WOM messages by people.

Drawing on the literature on psychological distance and construal levels of abstraction (Trope et al., 2007), we further speculate that people may perceive different types of norms (descriptive norms vs. injunctive norms) differently in terms of their psychological distance and level of abstraction. We suppose that injunctive norms, which usually serve as and are represented as a standard, are perceived as more distant than descriptive norms, which usually describe a particular behavior. Injunctive norms in the form of a standard typically describe a state of affairs that is expected to be realized in the future or in an ideal situation. Injunctive norms are thus more abstract than descriptive norms because inferences about the thoughts or opinions of others on an issue are typically more abstract than observations about the behaviors of others. Based on this, we argue that injunctive norms are perceived as more psychologically distant than descriptive norms and at a higher level of construal than descriptive norms.

On the other hand, prior research on norms and its impact on consumer choices has been exclusively focused on descriptive norms (Goldstein et al., 2008; Zhao and Xie, 2011; Ryoo et al., 2017). Little research has examined the difference between injunctive norms and descriptive norms, especially regarding their impact on the WOM communication behavior of consumers.

To fill the gap and examine the underlying process of the different impacts of descriptive vs. injunctive norms, this study investigated the interaction effect of social group distance with the type of norms associated with the group and its impact on WOM communication. According to the Fit Theory (Higgins, 2000; Higgins et al., 2003), the congruency between the external stimulus (e.g., the message frame) and the internal mind-set (e.g., construal level) of a consumer leads to greater persuasiveness of the message than when it is incongruent. Applying this general notion to the context of social group distance and norms type, we hypothesize that messages featuring group norms become more effective in motivating WOM when there is a match of construal level between the social group and norms type. Based on the argument on the different psychological distance of descriptive norms and injunctive norms aforementioned, we propose that the willingness of people to share messages featuring group norms will increase when there is a match of psychological distance of norms type (i.e., descriptive norms vs. injunctive norms) and the social group. We suppose that the consistency between a social group and the type of norms in terms of psychological distance should make people “feel right.” The congruency (feeling of fit) should in turn improve the willingness of the consumers to share the message. Specifically, we propose that the injunctive norms (vs. descriptive norms) of a distant group are more influential, whereas the descriptive norms of a close group (vs. injunctive norms) are more impactful in driving WOM. Based on this argument, we hypothesize that:

**H2**: The impact of a close group on WOM communication is larger than that of a distant group for descriptive norms compared with injunctive norms; whereas, the impact of a distant group on WOM communication is larger than that of a close group for injunctive norms compared with descriptive norms.

## Materials and Methods

### Study 1a: Group Norms and Word-Of-Mouth: Laboratory Experiment

The aim of Study 1a is to investigate the impact of group norms information in messages (news article) on WOM communication intention (i.e., sharing of the news article on Facebook). Would people be more willing to share a news article if it featured group norms than if without?

#### Materials and Methods

One hundred and seven (57.9% female) undergraduate students from a major university in the northeastern United States participated in the study voluntarily, without receiving extra class credits (this study was approved by University Institutional Review Board). Participants were randomly assigned to either the experimental or the control group. The experiment was a controlled (group norms vs. no group norms) between-subjects design. The social group of “scientists” was selected and associated with the descriptive norm of “consuming vegetarian meals at least twice per week” to form the featured group norms in a news article.

After reading the survey instructions, participants were then directed to read a news article about the benefits of consuming vegetarian dishes and statistics on vegetarian consumption patterns. The first part of the news article defined what a vegetarian meal is and described the health and environmental benefits of consuming vegetarian meals; the second part of the news article reported fabricated statistics attributed to the American Health Association. In the second half of the news article, experimental group participants were exposed to the following normative information: 60% of scientists reported that they consume (descriptive norms) vegetarian meals at least twice per week. In comparison, participants in the control group were directed to read fabricated statistics of the average vegetarian consumption in general instead.

After reading the news article, participants were directed to answer two questions to indicate their WOM intentions. eWOM: “How likely would you be to share this news article on your Facebook (1 = Not at all likely; 7 = Very likely)?”; fWOM: “How likely would you be to recommend the article to your family and friends when you meet them in person (1 = Not at all likely; 7 = Very likely)?”

#### Results

Independent sample *t*-tests revealed a statistically significant main effect of group norms on eWOM (*t*(105) = 2.20, *p* = 0.03, Cohen's *d* = 0.43, 95% CI = [0.04, 0.82]). The message with group norms (*M* = 2.86, SD = 1.71) was significantly more effective in motivating eWOM than the control group (*M* = 2.16, SD = 1.58; *p* = 0.03). Furthermore, the main effect of group norms on fWOM was marginally significant (*t*(105) = 1.9, *p* = 0.06, Cohen's *d* = 0.43). The message with group norms (*M* = 3.32, *SD* = 1.80) was more effective in motivating fWOM than the control group (*M* = 2.67, *SD* = 1.70).

#### Discussion

Study 1a suggests that messages featuring group norms (descriptive norms) are more likely to be shared on social media than messages with no normative information, and it provides preliminary support for the hypothesis that normative cues can drive WOM communication. Descriptive norms were tested in Study 1a since they are typically perceived as more impactful and prevalent than injunctive norms. To further examine the impact of normative cues on WOM communication in a realistic setting, Study 1b was conducted to test WOM communication of messages of the consumers with group norms on social media.

### Study 1b: Group Norms and Word-Of-Mouth: A Field Study

The group norms information in Study 1a involved a special group with relatively high social status and credibility, scientists. In addition, the normative behavior was the consumption of vegetarian meals, which is a prosocial sustainable behavior. To remove the potential confounding effect of the social desirability and prosocial characteristics of group norms in Study 1a, Study 1b chose a more neutral group, which is a Chinese middle-aged group. Leveraging the attention of the general public on COVID-19 in the spring of 2020, Study 1b examined the sharing behavior of messages containing group norms such as “Chinese middle-aged people do not like wearing face masks when going out” which became a hot topic online during the early stage of the COVID-19 epidemic in China.

#### Materials and Methods

Data were collected from Sina Weibo, one of the largest Chinese microblogging websites. With keywords such as “60s generation + do not wear face mask” or “60s generation + coronavirus epidemic,” a total of 1,616 fuzzy matching microblogs were acquired using the Python crawler. Microblogs were then screened with only original microblogs kept for further studies. The microblogs were then evaluated by two independent research assistants to determine if the content of the microblogs met the following criteria: (1) microblogs with group norms (e.g., “the generation of those in their 60s do not wear face masks when going out. How can they be persuaded to do so?”). To qualify, the salient normative information needed to include “most people in their 60s do not wear face masks” in the message; the microblog could only involve one subject, (those in their 60s), any microblog involving individual persons or other generations such as those in their 80s or 90s would be excluded. All three criteria had to be met simultaneously. (2) Microblogs without group norms (e.g., “the generation of those in their 60s should take good care of themselves during the COVID-19 outbreak, the following are some tips to keep healthy…”). To be included, the generation of those in their 60s had to be the subject of the microblog; the microblog had to contain information about the coronavirus epidemic; the microblog could not contain any normative information related to the social group of the generation of those in their 60s. All three criteria had to be met simultaneously. After screening, 47 microblogs meeting the criteria were kept for further analyses.

Similar to Tik Tok and generation Z in the US, Sina Microblog is an online community that mainly attracts the young population (born in the 90s or 00s) of China. It is noteworthy that over 95% of Sina Weibo users were born on or after 1980 (Sina Weibo Data Center, 2019), and the average age of the users who repost the microblogs on the platform is 25 years (mostly 90s generation). This largely precludes the possibility that those in their 60's account for a large part of users who repost the microblogs because the topic or the norms involves people like themselves.

#### Results

Correlation analysis suggested a positive correlation between group norms (with vs. without) and eWOM, *r* = 0.29, *p* = 0.047. Microblogs with group norms (*M* = 623.70, *SD* = 1,378.81) had significantly higher repost numbers than microblogs without group norms (*M* = 7.05, *SD* = 9.97; *p* = 0.047).

We regressed eWOM (i.e., the number of reposts of each microblog) on the presence of group norms. The model was significant, *F*_(1, 47)_ = 4.18, *R*^2^ = 0.083, *p* = 0.047. Results showed a main effect for group norms, *b* = 616.66, *t* (47) = 2.04, *p* = 0.047. This suggested that when a microblog contained group normative information, the number of reposts of the microblog increased by 616.66 (i.e., over 616 more Weibo users shared the microblog on social media). With reference to normative information on WOM, Study 1b revealed that group normative information was positively correlated with WOM communication on social media.

#### Discussion

Using real repost data from a microblogging website, Study 1b showed that microblogs featuring normative cues (group norms) positively impacted the number of reposts of the microblog. Taken together, Studies 1a and 1b demonstrated a significant impact of group norms on eWOM both in the laboratory setting and in the field. To further investigate the impact of different types of norms (descriptive vs. injunctive norms) and its relationship with social groups on WOM communication, Study 2 was conducted to uncover the hypothesized interaction effect.

### Study 2

Study 2 tested the role of the psychological distance of reference groups in relationship with different types of norms on WOM communication. We examined whether a close group (vs. a distant group) would be more impactful in motivating WOM communication of a news article featuring group norms, accounting for a different type of norms.

#### Materials and Methods

Two hundred and seventy-five college students (55.4% male) from a major northeastern university in the United States participated in the research for class credit. Participants were assigned randomly to one of the five conditions in an incomplete 2 (social group: close group vs. distant group) x 2 (norms type: descriptive norms vs. injunctive norms) plus a control group (no group norms information) between-subjects experimental design.

As the first part of the study, participants who did not have Facebook accounts were screened out. Participants who enrolled in the study were then asked to imagine reading a news article online. Similar to Study 1a, the first part of the article defined a vegetarian diet and described the health and environmental benefits of being on a vegetarian diet, and the second part of the news article reported fabricated statistics attributed to the American Health Association. In the second half, it was reported that 60% of the social group is consuming (descriptive group norms) or thought they should have (injunctive group norms) consumed vegetarian meals at least twice per week. This was followed by a comment that the prevalence of vegetarian consumption in this social group is 50% higher than that of average college students in the United States. The article ended by stating the reasons why the social group chose a vegetarian dish, 70% chose it for environmental concerns and 30% for both nutrition and environmental concerns.

#### Operationalization of Reference Group Distance

Operationalization of reference group distance was based on the school affiliations of the participants. One should feel naturally close to students studying within the same university but feel more distant to students studying at a different university. Thus, in the close group condition, participants read news articles associating vegetarian consumption with students from the same university, and in the distant group condition, participants read a news article associating vegetarian consumption with students from a different university. In the control group, no social group norms information was included.

#### Outcome Measurements: WOM Intentions

After reading the news article, participants were directed to answer two questions to indicate WOM intentions. eWOM: “How likely would you be to share this news article on your Facebook (1 = Not at all likely; 7 = Very likely)?” fWOM: “How likely would you be to recommend the article to your family and friends when you meet them in person (1 = Not at all likely; 7 = Very likely)?” After screening out outliers, we retained 240 participants in total. Two-way ANOVAs were conducted to analyze the main effects and interaction items on WOM intentions.

#### Results

**Figure 1:**
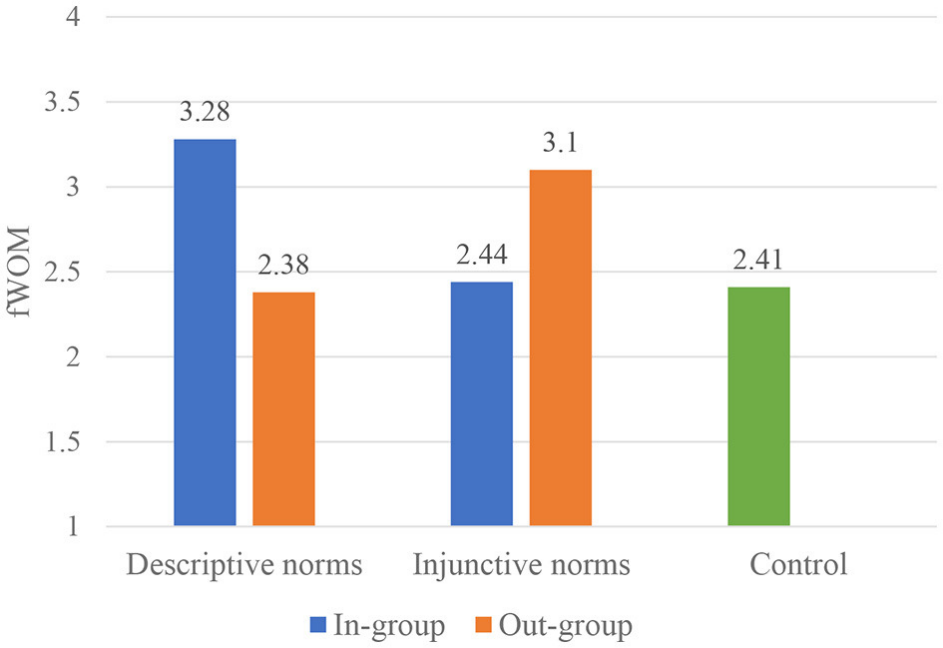
The interaction of social group and norms type on fWOM (Study 2).

Two-way ANOVAs found no significant main effects of either social group or norms type, but a significant interaction of group distance by norms type on fWOM was revealed (*F*_(1, 240)_ = 7.64, *p* < 0.01, $\eta_{p}^{2}$= 0.03, 95% CI = [0.00, 0.08]). *Post hoc* analysis (LSD), following a one-way ANOVA, was conducted to make follow-up comparisons. As illustrated in Figure 1, the results showed that close group descriptive norms are significantly more influential (*M* = 3.28) in motivating people to engage in fWOM than distant group descriptive norms (*M* = 2.38; *p* = 0.02) and the control group (*M* = 2.41; *p* = 0.02); distant group injunctive norms are marginally significantly more influential (*M* = 3.10) in motivating people to engage in fWOM than the control group (*M* = 2.41; *p* < 0.1), although no significant differences were found between close group and distant group injunctive norms on fWOM. No significant interaction effect was found on eWOM.

#### Discussion

Study 2 examined the interaction effect of social group distance with norms type on WOM intentions. A significant interaction was found, suggesting the greater motivating effect of group norms when a psychological distance of the social group and the type of norms are congruent. News articles featuring distant group injunctive norms did not differ from those featuring close group injunctive norms in terms of motivating WOM but were significantly more likely to be shared than in the control condition without group norms information. It is assumed that although the impact of close group injunctive norms on WOM may be diminished due to the incongruence of psychological distance between a close group and injunctive norms, the in-group identity might moderate the decreased effect, leading to non-significant differences between the two conditions. It has been shown that sharing a particular subject matter online creates a feeling of group identity and fosters the aggregate sense of an individual of his/her self (Belk, 2013). Thus, the non-significant difference between close group injunctive norms and distant group injunctive norms might be due to the confounding impact of group identity on WOM.

### Study 3

Study 3 employed two social groups with different social distance so as to remove the confounding effect of social identity on the impact of social distance on WOM communication in Study 2. Study 3 tried to examine whether social distance could exert its influence on WOM independent of the effect of social identity.

#### Materials and Methods

Two hundred and forty-seven (56.5% female) undergraduate students from a major university in the northeastern United States participated in the study for class credits. Participants were randomly assigned to one of five conditions in an incomplete 2 (social group: close vs. distant) x 2 (norms type: descriptive vs. injunctive) plus a control group between-subjects design.

##### Operationalization of Reference Group Distance

Perceived social distance of reference groups was operationalized based on the next-step career planning of the participants (i.e., what kind of people they want to become after finishing college). Participants were asked to choose from the following options about their career plans immediately after graduation from college: (1) go to graduate school, (2) become a working professional. After making the choice, they were instructed to answer five social distance measurement questions adapted from Kim et al. (2008), which was as follows (1) for participants who chose “go to graduate school” in the previous question: “How important it is to you to be seen as someone who aspires to pursue an advanced degree (Masters, PhD, or JD, etc.)?”; for participants who chose “become a working professional” in the previous question: “How important it is to you to be seen as someone who aspires to pursue a successful professional career)?” (1 = Not at all important; 7 = Very important); (2) and (3) “How easily can you picture yourself becoming a graduate student? or a working professional?” (1 = Not at all easily; 7 = Very easily); (4) and (5) “How similar do you think you are compared with graduate students in general or working professionals in general?” (1 = Not at all similar; 7 = Very similar).

##### Group Condition Check

We averaged the responses of the participants to the five questions on the perceived social distance of the reference group to form a social distance score (SDS; α = 0.60; the smaller the score, the closer the social distance). One-way ANOVAs, looking at the effects of the career plans of participants after college on SDS toward the two social groups, revealed the following: (1) Participants who planned to go to graduate school perceived graduate students as more similar and less socially distant to themselves (*M* = 2.61) than working professionals [*M* = 4.95, *F*_(1, 246)_ = 233.23, *p* < 0.00); (2) participants who planned to become working professionals after college perceived working professionals as more similar and less socially distant to themselves (*M* = 2.18) than graduate students [*M* = 4.28, *F*_(1, 246)_ = 245.75, *p* < 0.001].

#### Results

**Figure 2:**
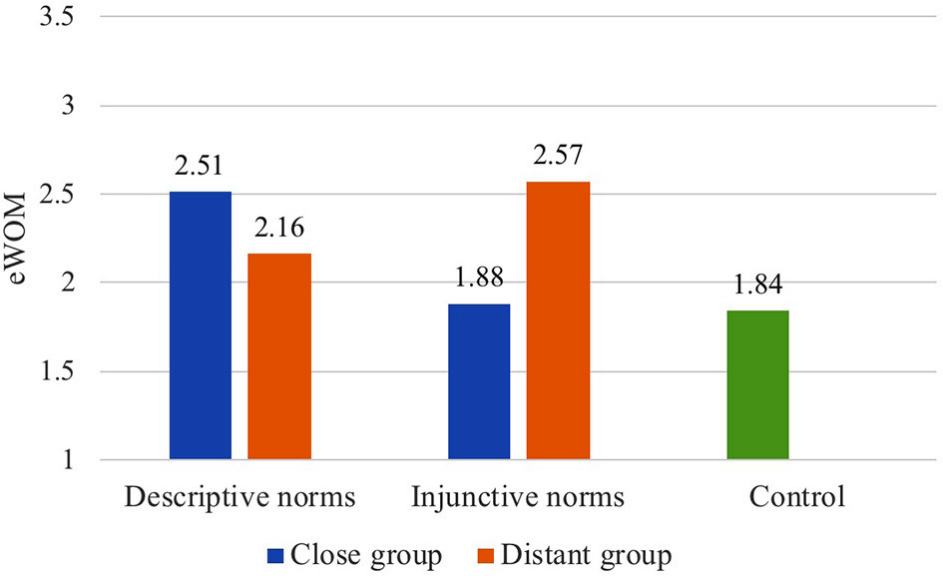
The interaction of group distance and norms type on eWOM (Study 3).

**Figure 3:**
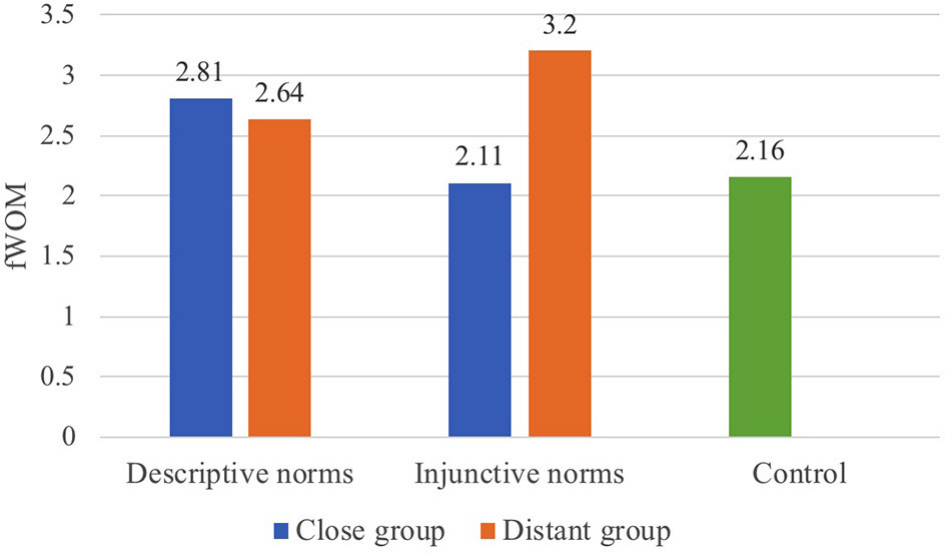
The interaction of group distance and norms type on fWOM (Study 3).

Two-way ANOVAs showed no significant main effects but revealed statistically significant interactions between group distance and norms type on eWOM (*F*_(1, 246)_ = 4.96, *p* = 0.03, $\eta_{p}^{2}$= 0.02, 95% CI = [0.00, 0.07]; see Figure 2) and fWOM (*F*_(1, 246)_ = 4.81, *p* = 0.03, $\eta_{p}^{2}$= 0.02, 95% CI = [0.00, 0.07]; see Figure 3).

As illustrated in Figure 2, *post-hoc* analysis (LSD) following one-way ANOVA on eWOM showed that distant group injunctive norms (*M* = 2.57) are significantly more effective in motivating eWOM than close group injunctive norms (*M* = 1.88; *p* = 0.05) and the control group (*M* = 1.84; *p* = 0.04). In addition, close group descriptive norms (*M* = 2.51) are significantly more effective in driving eWOM than the control group (*M* = 1.84; *p* = 0.04), although no significant differences were found between close group and distant group descriptive norms on eWOM.

As illustrated in Figure 3, *post-hoc* analysis (LSD), following one-way ANOVA, on fWOM showed that distant group injunctive norms (*M* = 3.2) are significantly more effective in motivating fWOM than close group injunctive norms (*M* = 2.11; *p* = 0.01) and the control group (*M* = 2.16; *p* < 0.01); close group descriptive norms (*M* = 2.81) are marginally more influential in motivating people to engage in fWOM than the control group (*M* = 2.16; *p* = 0.07), although no significant differences were found between close group and distant group descriptive norms on fWOM. No significant differences were found among other conditions.

#### Discussion

Complementing Study 2, Study 3 confirmed that group distance, independent of social identity, does interact with norms type when motivating WOM communication. The impacts of distant group injunctive norms on both eWOM and fWOM are significantly more influential than close group injunctive norms. This confirmed the hypothesis that the psychological distance congruency of the distant group and injunctive norms contained in the messages makes people more willing to share the message. Although close group descriptive norms were not significantly more impactful than distant group descriptive norms in terms of motivating WOM communication, a similar interaction pattern as in Study 2 between group distance and norms type was found in Study 3.

## General Discussion

This study examined the impact of group norms on WOM intention from a psychological distance perspective and investigated the interaction of group distance with norms type on WOM. Overall, the results suggested that normative cues in the message can effectively drive WOM communication, and the effect varies by the congruence of psychological distance between the social group and norms type (descriptive vs. injunctive) tied to the group.

As illustrated in Table 1, Study 1a and Study 1b showed that messages featuring group norms were more likely to be shared on social media (eWOM) both in a laboratory setting and in the field. Study 2 examined the interaction effect between group distance and norms type. The results showed a greater impact of in-group descriptive norms on WOM than that of out-group descriptive norms, but no significant difference was found between in-group injunctive norms and out-group injunctive norms. We assume this might be due to the positive impact of in-group identification in motivating WOM. Prior research showed that in-group norms exert influence through accentuating feelings of group belongingness, which makes one align their perceptions, beliefs, attitudes, and behaviors with the in-group norms (Terry and Hogg, 1996). People might be more willing to share messages featuring in-group norms because doing so would help strengthen their sense of group belongingness and identity. This might enhance the impact of out-group injunctive norms on WOM in comparison to in-group injunctive norms.

**Table 1:** Results summary of Study 1–3.

	**eWOM**	**fWOM**
Study 1a	Message with group norms > Message without group norms **(H1)**	Message with group norms > Message without group norms **(H1)**
Study 1b (Field Study)	A positive correlation between group norms (with vs. without) and eWOM **(H1)**	N/A
Study 2	No significant difference	In-group descriptive norms > Distant group descriptive norms = control group **(H2)**In-group injunctive norms = Out-group injunctive norms > control group
Study 3	Close group descriptive norms > Distant group descriptive norms = control group **(H2)** Distant group injunctive norms > Close group injunctive norms = control group **(H2)**	Close group descriptive norms = Distant group descriptive norms > control group Distant group injunctive norms > Close group injunctive norms = control group (H2)

To remove the potential confounding effect of group identity on WOM communication, Study 3 employed two out-groups with different social distances to examine its relationship with norms type on WOM communication. The results showed that distant group injunctive norms were more effective in driving eWOM and fWOM than were close group injunctive norms. We assume that the psychological distance congruency of the distant group and injunctive norms increases the perceived effectiveness or credibility of the message, such that people are more willing to share it on social media or in person. Although no significant difference was found between close group descriptive norms and distant group injunctive norms, the interaction pattern between the two groups was consistent with that in Study 2. Close group descriptive norms were marginally more effective in motivating eWOM and fWOM than in the control group, which echoes the findings in Studies 1a and 1b.

In terms of the effects of group norms on eWOM and fWOM, the absence of interaction effects on eWOM in Study 2 suggests that people maybe more cautious with eWOM than with fWOM. This was consistent with the finding by Eisingerich et al. (2015) that consumers are less willing to engage in online WOM for the fear of higher social risks associated with WOM on SNS than those associated with in-person situations. Given the mixed results in terms of descriptive vs. injunctive impact of norms on eWOM/fWOM, we suspect that there may be several factors that influence WOM communication intention in addition to psychological distance congruence between norms type and social groups. For example, the target audience of eWOM vs. fWOM differs in quantity, closeness, and importance. Sharing a piece of news with family and friends of an individual means WOM among a small group of (perhaps 3–8) relatively close and important people, whereas sharing a piece of news on social media (e.g., Facebook) is the equivalent of broadcasting to a large number of people, the majority of whom you may not know well (or at all). It is highly possible that the increased social risk and social cost of eWOM make participants less willing to share on social media, especially when the piece of news involves an in-group (Study 2). This could possibly explain the non-significant impact of group norms on eWOM in Study 2. Overall, the effect of normative cues on both eWOM and fWOM was shown to be significant across social groups with various social distances and different norms type.

### Theoretical Contribution

This study offers several theoretical contributions. First, to the best of our knowledge, this is the first research to examine the impact of different types of group norms from a psychological distance perspective. It is also the first to uncover the interaction effect of group distance with norms type and their impact on WOM communication. Prior research has suggested that provincial descriptive norms are more impactful than global descriptive norms in persuading people to engage in sustainable consumption (Goldstein et al., 2008; Ryoo et al., 2017). Furthermore, recent research has shown that descriptive norms have a greater impact on the own decisions of a person, whereas injunctive norms have a greater influence on recommendations to others (Zou and Savani, 2019). Based on these findings, this study proposes and finds support that psychological distance congruency plays a significant role in influencing the impact of norms on persuasion. This study takes advantage of the different psychological distances of social groups coupled with either descriptive or injunctive norms and uncovered the interaction effect between group distance and norms type. This finding further contributes to the understanding of the distinctive nature of descriptive vs. injunctive norms in terms of the perceived psychological distance and their impact on WOM communication.

Second, this study extends the fit literature (e.g., Higgins, 2000; Higgins et al., 2003; Kim et al., 2009), adding the dimension of the psychological distance of group norms. The findings of this study suggest that messages with normative cues become more effective in motivating WOM when there is a match of construal levels between the psychological distance of social group and norms type. Based on the fit theory (Higgins, 2000; Higgins et al., 2003), we believe that the congruence between social group and norms type in terms of their psychological distance and construal level should make people “feel right.” The feeling of “fit” further improves the WOM communication behavior of the consumers. Specifically, since a distant group was construed at a higher level (vs. a close group), which is congruent with a higher construal level of people for injunctive norms (vs. descriptive norms), the distant group had a greater effect on WOM communication when incorporating injunctive norms than descriptive norms.

Finally, this study demonstrates that normative cues contained in the message can drive WOM communication of the message. Prior research has identified a large set of factors that drive WOM, including whether the message content is interesting, novel, surprising, useful, practical, or even controversial (Bakshy et al., 2011; Berger and Milkman, 2012; Chen and Berger, 2013). However, research on the influence of normative cues in motivating WOM remains unclear. This study suggests that news articles or microblogs featuring group norms are more likely to be shared than those without. This extends this study on motivators of WOM communication.

### Practical Implications

This study tries to bring the practical values of group norms to the wider attention of the field of social media communication. The findings on the influence of normative cues on eWOM inform the data mining strategies of digital marketers designed to create effective algorithms to identify the right group of people to endorse certain products. Celebrity endorsement has had declining impacts on social media (Smith et al., 2007), and microinfluencers also have their limits in reaching out to a broad population. This study suggests an alternative option, which is using a customized reference group that the target audience can easily relate to. Our findings suggest that the norms type employed in the message should be congruent with the psychological distance of the selected social group so as to make the message more likely to be shared among the target audience on social media.

### Limitations and Future Research

One primary limitation of the study is the intricate conceptualization of social identity and perceived social distances. It is noteworthy that considerable perceived social distance should exist between the in-group and out-group in Study 1. We cannot rule out the possibility that the perceived social distance difference might explain part of the interaction in addition to social identity in Study 2. Therefore, we conducted another study (Study 3) to test social distance separately. Although the comparison between Studies 2 and 3 provides some insights on the issue, future studies should identify a way to separate social identity from a social distance. Second, the reference groups selected in the studies are naturally occurring social groups. We believe using naturally occurring groups has a practical value, considering that people do self-select into different social groups in the real world. Future research should be conducted to see if the results are replicable when manipulating reference group conditions in a stricter way.

## Data Availability Statement

The raw data supporting the conclusions of this article will be made available by the authors, without undue reservation.

## Ethics Statement

The studies involving human participants were reviewed and approved by Beverly Tepper, Ph.D. IRB Chair, Arts and Sciences Institutional Review Board, Rutgers, The State University of New Jersey. The patients/participants provided their written informed consent to participate in this study.

## Author Contributions

MZ, WH, and JL contributed to the conception, design of the study, and wrote sections of the manuscript. MZ organized the database and performed the statistical analysis. All authors contributed to manuscript revision, read, and approved the submitted version.

## Funding

This work was supported by the Fundamental Research Funds for the Central Universities [Grant Nos. 2019ECNU-HWFW027 and 2018ECNU-HLYT027], Shanghai Pujiang Program [Grant No. 2019PJC036], Shanghai Philosophy and Social Sciences Research Program [Grant No. 2020JG003-EGL194], and the National Natural Science Foundation of China [Grant Nos. 71771088 and 72102074].

## Conflict of Interest

The authors declare that the research was conducted in the absence of any commercial or financial relationships that could be construed as a potential conflict of interest.

## Publisher's Note

All claims expressed in this article are solely those of the authors and do not necessarily represent those of their affiliated organizations, or those of the publisher, the editors and the reviewers. Any product that may be evaluated in this article, or claim that may be made by its manufacturer, is not guaranteed or endorsed by the publisher.
